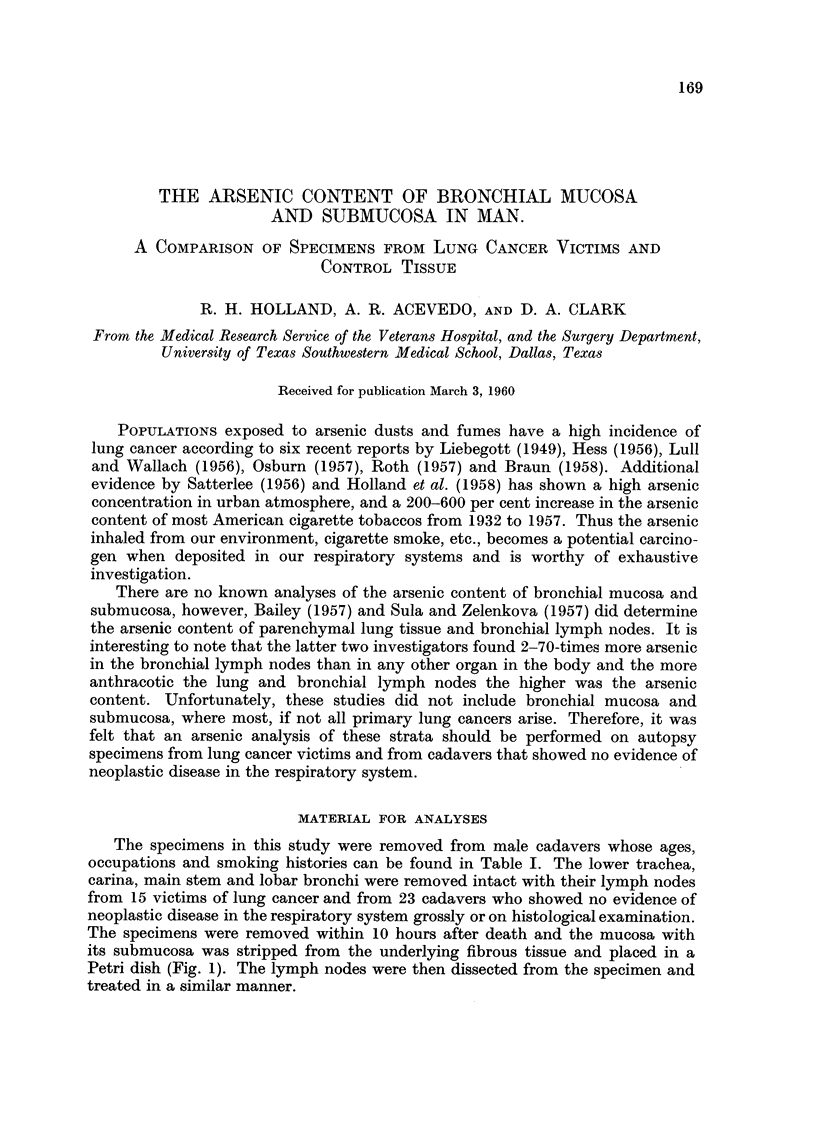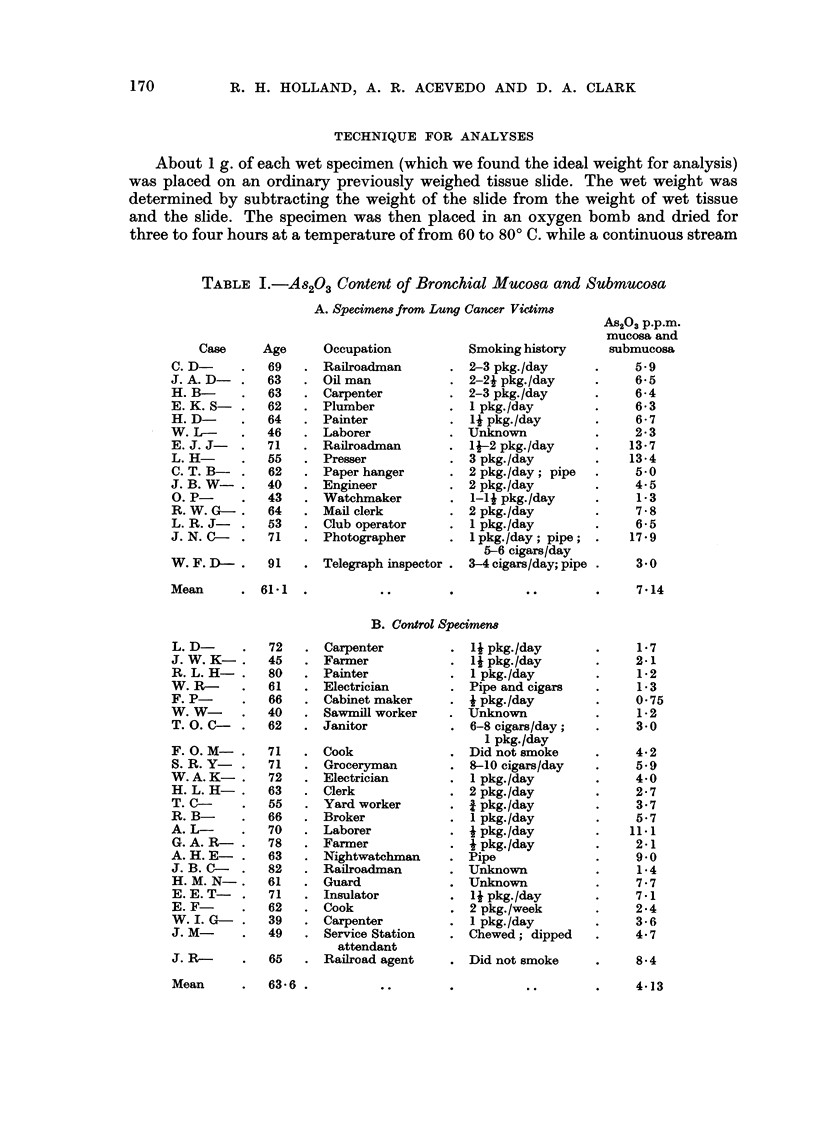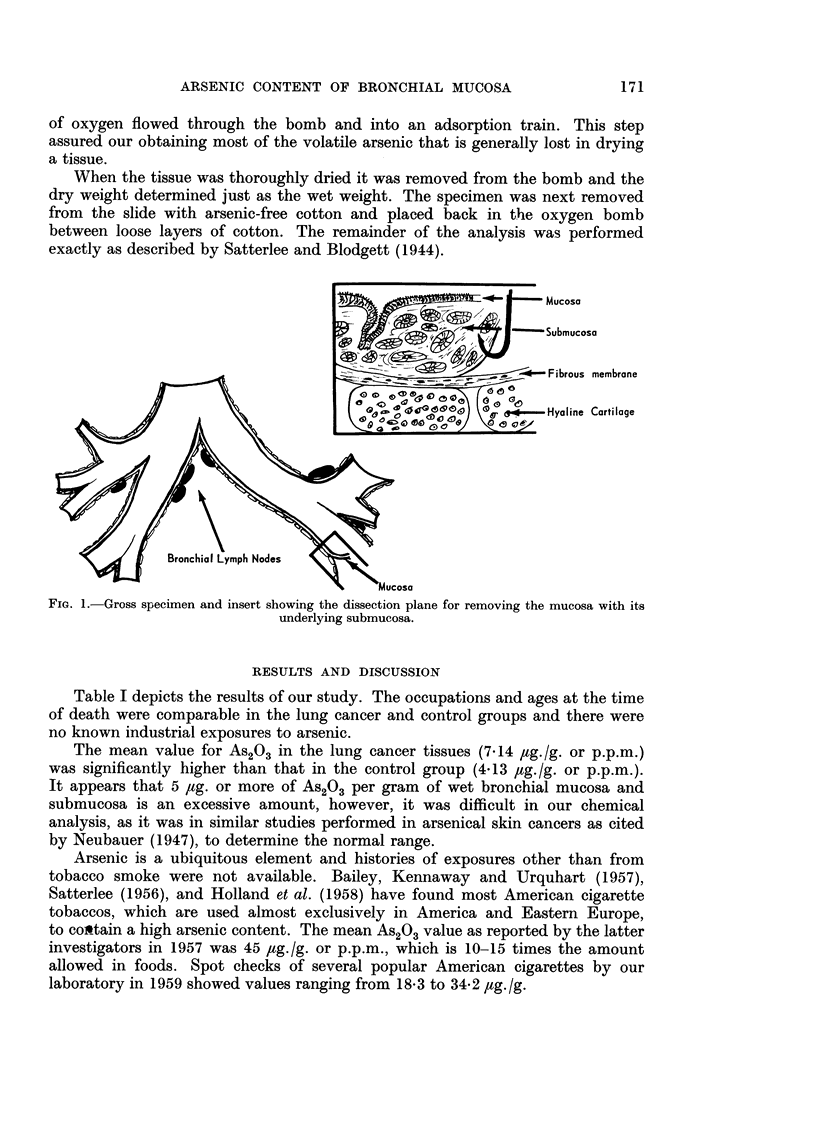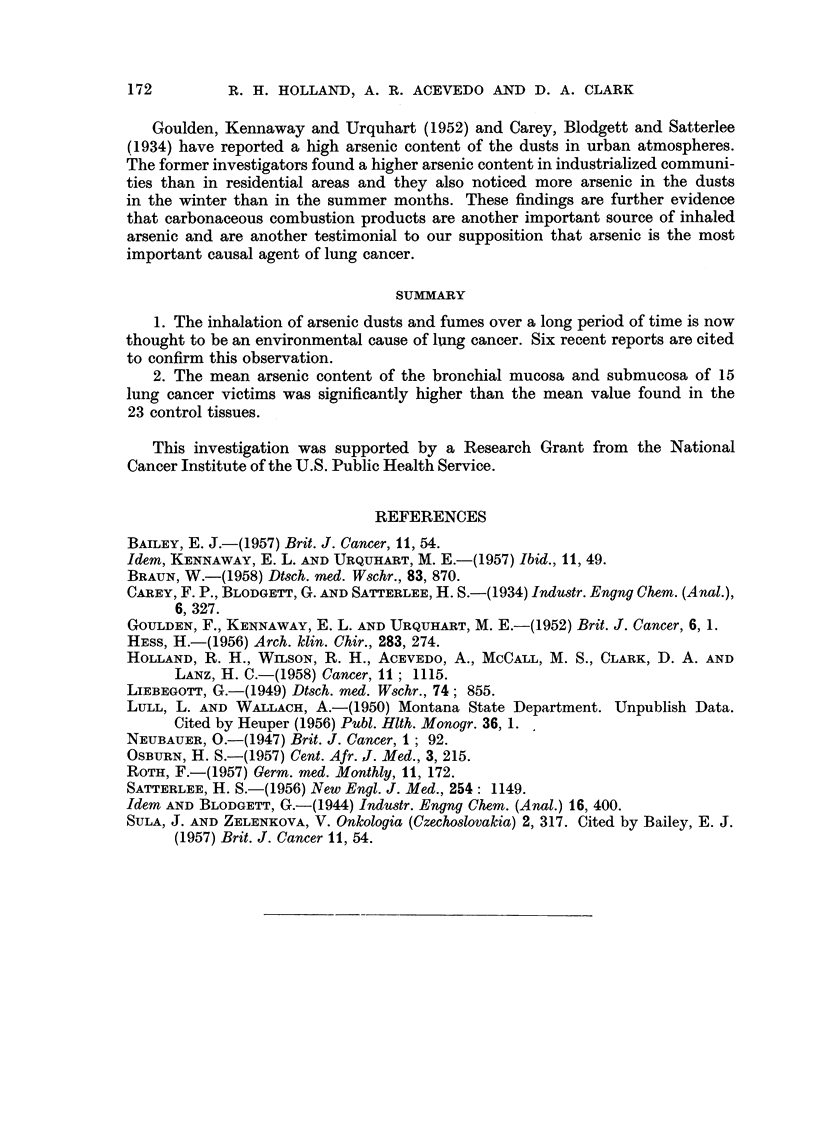# The Arsenic Content of Bronchial Mucosa and Submucosa in Man

**DOI:** 10.1038/bjc.1960.20

**Published:** 1960-06

**Authors:** R. H. Holland, A. R. Acevedo, D. A. Clark


### THE ARSENIC CONTENT OF BRONCHIAL MUCOSA AND SUBMUCOSA IN MAN.

### A Comparison of Specimens from Lung Cancer Victims and Control Tissue

R. H. HOLLAND, A. R. ACEVEDO, and D. A. CLARK

*From the Medical Research Service of the Veterans Hospital, and the Surgery Department, University of Texas Southwestern Medical School, Dallas, Texas*

Received for publication March 3, 1960

Populations exposed to arsenic dusts and fumes have a high incidence of lung cancer according to six recent reports by Liebegott (1949), Hess (1956), Lull and Wallach (1956), Osburn (1957), Roth (1957) and Braun (1958). Additional evidence by Satterlee (1956) and Holland *et al.* (1958) has shown a high arsenic concentration in urban atmosphere, and a 200–600 per cent increase in the arsenic content of most American cigarette tobaccos from 1932 to 1957. Thus the arsenic inhaled from our environment, cigarette smoke, etc., becomes a potential carcinogen when deposited in our respiratory systems and is worthy of exhaustive investigation.

There are no known analyses of the arsenic content of bronchial mucosa and submucosa, however, Bailey (1957) and Sula and Zelenkova (1957) did determine the arsenic content of parenchymal lung tissue and bronchial lymph nodes. It is interesting to note that the latter two investigators found 2–70-times more arsenic in the bronchial lymph nodes than in any other organ in the body and the more anthracotic the lung and bronchial lymph nodes the higher was the arsenic content. Unfortunately, these studies did not include bronchial mucosa and submucosa, where most, if not all primary lung cancers arise. Therefore, it was felt that an arsenic analysis of these strata should be performed on autopsy specimens from lung cancer victims and from cadavers that showed no evidence of neoplastic disease in the respiratory system.

### MATERIAL FOR ANALYSES

The specimens in this study were removed from male cadavers whose ages, occupations and smoking histories can be found in Table I. The lower trachea, carina, main stem and lobar bronchi were removed intact with their lymph nodes from 15 victims of lung cancer and from 23 cadavers who showed no evidence of neoplastic disease in the respiratory system grossly or on histological examination. The specimens were removed within 10 hours after death and the mucosa with its submucosa was stripped from the underlying fibrous tissue and placed in a Petri dish (Fig. 1). The lymph nodes were then dissected from the specimen and treated in a similar manner.

### TECHNIQUE FOR ANALYSES

About 1 g. of each wet specimen (which we found the ideal weight for analysis) was placed on an ordinary previously weighed tissue slide. The wet weight was determined by subtracting the weight of the slide from the weight of wet tissue and the slide. The specimen was then placed in an oxygen bomb and dried for three to four hours at a temperature of from 60 to 80° C. while a continuous stream

TABLE I.—*$As_2O_3$ Content of Bronchial Mucosa and Submucosa*

| Case | Age | Occupation | Smoking history | $As_2O_3$ p.p.m. mucosa and submucosa |
|---|---|---|---|---|
| A. *Specimens from Lung Cancer Victims* | | | | |
| C. D— | 69 | Railroadman | 2–3 pkg./day | 5·9 |
| J. A. D— | 63 | Oil man | 2–2½ pkg./day | 6·5 |
| H. B— | 63 | Carpenter | 2–3 pkg./day | 6·4 |
| E. K. S— | 62 | Plumber | 1 pkg./day | 6·3 |
| H. D— | 64 | Painter | 1½ pkg./day | 6·7 |
| W. L— | 46 | Laborer | Unknown | 2·3 |
| E. J. J— | 71 | Railroadman | 1½–2 pkg./day | 13·7 |
| L. H— | 55 | Presser | 3 pkg./day | 13·4 |
| C. T. B— | 62 | Paper hanger | 2 pkg./day ; pipe | 5·0 |
| J. B. W— | 40 | Engineer | 2 pkg./day | 4·5 |
| O. P— | 43 | Watchmaker | 1–1½ pkg./day | 1·3 |
| R. W. G— | 64 | Mail clerk | 2 pkg./day | 7·8 |
| L. R. J— | 53 | Club operator | 1 pkg./day | 6·5 |
| J. N. C— | 71 | Photographer | 1 pkg./day ; pipe ; 5–6 cigars/day | 17·9 |
| W. F. D— | 91 | Telegraph inspector | 3–4 cigars/day; pipe | 3·0 |
| Mean | 61·1 | .. | .. | 7·14 |
| B. *Control Specimens* | | | | |
| L. D— | 72 | Carpenter | 1½ pkg./day | 1·7 |
| J. W. K— | 45 | Farmer | 1½ pkg./day | 2·1 |
| R. L. H— | 80 | Painter | 1 pkg./day | 1·2 |
| W. R— | 61 | Electrician | Pipe and cigars | 1·3 |
| F. P— | 66 | Cabinet maker | ½ pkg./day | 0·75 |
| W. W— | 40 | Sawmill worker | Unknown | 1·2 |
| T. O. C— | 62 | Janitor | 6–8 cigars/day ; 1 pkg./day | 3·0 |
| F. O. M— | 71 | Cook | Did not smoke | 4·2 |
| S. R. Y— | 71 | Groceryman | 8–10 cigars/day | 5·9 |
| W. A. K— | 72 | Electrician | 1 pkg./day | 4·0 |
| H. L. H— | 63 | Clerk | 2 pkg./day | 2·7 |
| T. C— | 55 | Yard worker | ¾ pkg./day | 3·7 |
| R. B— | 66 | Broker | 1 pkg./day | 5·7 |
| A. L— | 70 | Laborer | ½ pkg./day | 11·1 |
| G. A. R— | 78 | Farmer | ½ pkg./day | 2·1 |
| A. H. E— | 63 | Nightwatchman | Pipe | 9·0 |
| J. B. C— | 82 | Railroadman | Unknown | 1·4 |
| H. M. N— | 61 | Guard | Unknown | 7·7 |
| E. E. T— | 71 | Insulator | 1½ pkg./day | 7·1 |
| E. F— | 62 | Cook | 2 pkg./week | 2·4 |
| W. I. G— | 39 | Carpenter | 1 pkg./day | 3·6 |
| J. M— | 49 | Service Station attendant | Chewed ; dipped | 4·7 |
| J. R— | 65 | Railroad agent | Did not smoke | 8·4 |
| Mean | 63·6 | .. | .. | 4·13 |

of oxygen flowed through the bomb and into an adsorption train. This step assured our obtaining most of the volatile arsenic that is generally lost in drying a tissue.

When the tissue was thoroughly dried it was removed from the bomb and the dry weight determined just as the wet weight. The specimen was next removed from the slide with arsenic-free cotton and placed back in the oxygen bomb between loose layers of cotton. The remainder of the analysis was performed exactly as described by Satterlee and Blodgett (1944).

FIG. 1.—Gross specimen and insert showing the dissection plane for removing the mucosa with its underlying submucosa.

### RESULTS AND DISCUSSION

Table I depicts the results of our study. The occupations and ages at the time of death were comparable in the lung cancer and control groups and there were no known industrial exposures to arsenic.

The mean value for $As_2O_3$ in the lung cancer tissues (7·14 μg./g. or p.p.m.) was significantly higher than that in the control group (4·13 μg./g. or p.p.m.). It appears that 5 μg. or more of $As_2O_3$ per gram of wet bronchial mucosa and submucosa is an excessive amount, however, it was difficult in our chemical analysis, as it was in similar studies performed in arsenical skin cancers as cited by Neubauer (1947), to determine the normal range.

Arsenic is a ubiquitous element and histories of exposures other than from tobacco smoke were not available. Bailey, Kennaway and Urquhart (1957), Satterlee (1956), and Holland *et al.* (1958) have found most American cigarette tobaccos, which are used almost exclusively in America and Eastern Europe, to contain a high arsenic content. The mean $As_2O_3$ value as reported by the latter investigators in 1957 was 45 μg./g. or p.p.m., which is 10–15 times the amount allowed in foods. Spot checks of several popular American cigarettes by our laboratory in 1959 showed values ranging from 18·3 to 34·2 μg./g.

Goulden, Kennaway and Urquhart (1952) and Carey, Blodgett and Satterlee (1934) have reported a high arsenic content of the dusts in urban atmospheres. The former investigators found a higher arsenic content in industrialized communities than in residential areas and they also noticed more arsenic in the dusts in the winter than in the summer months. These findings are further evidence that carbonaceous combustion products are another important source of inhaled arsenic and are another testimonial to our supposition that arsenic is the most important causal agent of lung cancer.

### SUMMARY

1. The inhalation of arsenic dusts and fumes over a long period of time is now thought to be an environmental cause of lung cancer. Six recent reports are cited to confirm this observation.

2. The mean arsenic content of the bronchial mucosa and submucosa of 15 lung cancer victims was significantly higher than the mean value found in the 23 control tissues.

This investigation was supported by a Research Grant from the National Cancer Institute of the U.S. Public Health Service.

### REFERENCES

Bailey, E. J.—(1957) *Brit. J. Cancer*, **11**, 54.

*Idem*, Kennaway, E. L. and Urquhart, M. E.—(1957) *Ibid.*, **11**, 49.

Braun, W.—(1958) *Dtsch. med. Wschr.*, **83**, 870.

Carey, F. P., Blodgett, G. and Satterlee, H. S.—(1934) *Industr. Engng Chem.* (*Anal.*), **6**, 327.

Goulden, F., Kennaway, E. L. and Urquhart, M. E.—(1952) *Brit. J. Cancer*, **6**, 1.

Hess, H.—(1956) *Arch. klin. Chir.*, **283**, 274.

Holland, R. H., Wilson, R. H., Acevedo, A., McCall, M. S., Clark, D. A. and Lanz, H. C.—(1958) *Cancer*, **11** ; 1115.

Liebegott, G.—(1949) *Dtsch. med. Wschr.*, **74** ; 855.

Lull, L. and Wallach, A.—(1950) Montana State Department. Unpublish Data. Cited by Heuper (1956) *Publ. Hlth. Monogr.* **36**, 1.

Neubauer, O.—(1947) *Brit. J. Cancer*, **1** ; 92.

Osburn, H. S.—(1957) *Cent. Afr. J. Med.*, **3**, 215.

Roth, F.—(1957) *Germ. med. Monthly*, **11**, 172.

Satterlee, H. S.—(1956) *New Engl. J. Med.*, **254** : 1149.

*Idem* and Blodgett, G.—(1944) *Industr. Engng Chem.* (*Anal.*) **16**, 400.

Sula, J. and Zelenkova, V. *Onkologia* (*Czechoslovakia*) **2**, 317. Cited by Bailey, E. J. (1957) *Brit. J. Cancer* **11**, 54.